# Multicellular tumor spheroid models to explore cell cycle checkpoints in 3D

**DOI:** 10.1186/1471-2407-13-73

**Published:** 2013-02-08

**Authors:** Jennifer Laurent, Céline Frongia, Martine Cazales, Odile Mondesert, Bernard Ducommun, Valérie Lobjois

**Affiliations:** 1 Université de Toulouse; ITAV-USR3505, F-31106, Toulouse, France; 2 CNRS; ITAV-USR3505, F-31106, Toulouse, France; 3 CHU de Toulouse, F-31059, Toulouse, France; 4 ITAV - USR3505, Centre Pierre Potier, 1 place Pierre Potier, 50624, 31106, Toulouse Cedex 1, BP, France

**Keywords:** Spheroid, 3D, Cell cycle, Checkpoint, Fucci reporters

## Abstract

**Background:**

MultiCellular Tumor Spheroid (MCTS) mimics the organization of a tumor and is considered as an invaluable model to study cancer cell biology and to evaluate new antiproliferative drugs. Here we report how the characteristics of MCTS in association with new technological developments can be used to explore the regionalization and the activation of cell cycle checkpoints in 3D.

**Methods:**

Cell cycle and proliferation parameters were investigated in Capan-2 spheroids by immunofluorescence staining, EdU incorporation and using cells engineered to express Fucci-red and -green reporters.

**Results:**

We describe in details the changes in proliferation and cell cycle parameters during spheroid growth and regionalization. We report the kinetics and regionalized aspects of cell cycle arrest in response to checkpoint activation induced by EGF starvation, lovastatin treatment and etoposide-induced DNA damage.

**Conclusion:**

Our data present the power and the limitation of spheroids made of genetically modified cells to explore cell cycle checkpoints. This study paves the way for the investigation of molecular aspects and dynamic studies of the response to novel antiproliferative agents in 3D models.

## Background

Models that closely mimic human cancer are essential for the understanding of the intimate growth mechanisms and for the development of new treatments. MultiCellular Tumor Spheroid (MCTS) is a 3D model that accurately reproduces the organization of a microtumor, recapitulating cell-cell and cell-microenvironment interactions [1,2]. While growing, spheroids display a gradient of proliferating cells that are located in the outer cell-layers whereas the quiescent cells are located more centrally. This cell heterogeneity is similar to what is found in microregions of a tumor [2-4]. It is now widely accepted that MultiCellular Tumor Spheroids accurately reproduce the 3D architecture of solid tumors, filling the gap between monolayer cultured cells and animal models, and their interest as evaluation models for new anti-cancer strategies is increasingly recognized [4,5]. A number of reports indicate that evaluation performed with MCTS is rather predictive of the efficiency of new antitumor compounds in patients and has proven in numerous studies to be of tremendous interest in characterizing the effects of chemotherapeutics [6]. In that context, MCTS models are particularly interesting as they reproduce the multicellular resistance (MCR) associated to cell-cell interaction, low drug penetration, resistance of quiescent cells located in the deepest and hypoxic regions [5].

Cell cycle progression is driven by cyclin-CDK (cyclin-dependent kinases) complexes the activities of which being controlled by several activators and inhibitors including CDK inhibitors (CKI), the polo-like and Aurora kinases as well as the CDC25 phosphatases. In response to DNA injuries or to DNA replication or chromosomes segregation defects, signaling pathways, named checkpoints, are activated. This activation results in cell cycle arrest through direct or indirect CDK inhibition, thus allowing cells to repair these defects, thereby protecting cells from genomic instability [7,8]. Numerous proteins implicated in the control of the cell cycle progression have been found to be mutated or misregulated in human tumors [7-9]. Moreover, the function of checkpoint proteins and their regulators are often found disrupted in tumor cells. As a consequence, cell cycle deregulation is a common feature of human cancer [10] and cancer cells are frequently characterized by unscheduled proliferation and accumulation of genomic instability. New therapeutics strategies that aim at targeting the cell cycle machinery and its checkpoints are currently of a major interest. Cyclin-Dependent Kinases and their cyclin regulatory subunits, as well as other relevant cell cycle regulators have been the focus of intensive efforts to identify selective inhibitory compounds [9,11-14]. Another promising approach toward cell cycle regulatory mechanisms is to target cell cycle checkpoint that ensure maintenance of the genome integrity [15,16]. In line with this, as we recently reported, pancreatic ductal adenocarcinoma cell lines-derived MCTS can be used to evaluate the spatio-temporal effect of the combination between gemcitabine and a checkpoint kinase 1 inhibitor, CHIR-124 [17].

The use of MCTS in the evaluation and in the pre-clinical development of new compounds targeting the cell cycle and checkpoint machineries strengthen the need for a better knowledge of the proliferation parameters in such 3D models, and for the engineering of innovative new evaluation models that could provide additional information on the effect of the evaluated compounds on cell cycle progression in 3D. How are proliferation markers expression and distribution in the different cell cycle phases modulated in the different regions of a multicellular tumor spheroid remains poorly known.

In this study, we characterized the cell cycle parameters change in 3D by using pancreatic MCTS models engineered to express cell cycle fluorescent reporters. We report that these MCTS models allow monitoring the activation of cell cycle checkpoints in 3D, thus providing innovative strategies for the evaluation of new antitumor drugs targeting cell cycle regulation.

## Methods

### Cell culture

Capan-2 pancreatic cancer cells stably expressing the Fucci-G1 red or the Fucci-S/G2/M green were kindly provided by Dr P. Cordelier [17]. These cell lines were cultured in DMEM/F12 glutamax (Invitrogen, France) containing 10% FCS and penicillin/streptomycin in a humidified atmosphere of 5% CO2 at 37°C.

### Spheroid generation and culture

Spheroids were prepared as previously described [17]. Briefly, 1000 cells/well in DMEM/F12 (Invitrogen, France) supplemented with EGF (20 ng/ml, Invitrogen) and B27 (1x, Invitrogen) were distributed in poly-HEMA-coated 96-round bottom well plates. Plates were subjected to centrifugation (6 min, 800 g) and then placed in a humidified atmosphere of 5% CO2 at 37°C. To obtain quiescent spheroids, EGF was removed by washing 400 μm in diameter spheroids three times with media containing 10% FCS and then incubated with this media without EGF and B27 for 1-6 days.

### Immunofluorescence on frozen sections

Spheroids were fixed for 2-3 h with formalin (Sigma), then washed with PBS and stored at 4°C. After fixation, spheroids were processed for 5 μm frozen sections. Spheroids were incubated in 15% then 30% sucrose in PBS for 24 h at 4°C, and were then embedded in Tissue-Tek, (Sakura Finetek) before sectioning. Sections were incubated with antibodies directed against Ki67 (rabbit polyclonal, Santa Cruz, 1 μg/ml), phosphorylated-HistoneH3 (rabbit polyclonal, Upstate, 1 μg/ml), Cyclin A (mouse monoclonal, Abcam, 1/50), Cyclin B (mouse monoclonal, Abcam, 0,6 μg/ml), Cyclin D1 (rabbit monoclonal, Spring, 1/100) or Cyclin E (rabbit, monoclonal, Epitomics, 1/250) overnight at 4°C. After washes in PBS/Triton 0.1% v/v, the secondary antibody was applied (Alexa 488-anti-mouse or Alexa 594-anti-rabbit, Molecular Probes, 1/800, for 1 h at room temperature). DNA was stained using DAPI.

### EdU labelling of spheroids

EdU labelling was performed by using the Click-it® EdU Alexa Fluor® imaging kit (Molecular Probes). Briefly, EdU (5-ethynyl-2´-deoxyuridine) is a thymidine analog that is incorporated in DNA during DNA synthesis. EdU detection relies on a simple and quick click reaction, a copper-catalyzed covalent reaction between an azide and an alkyne, that do not necessitate a DNA denaturation step like BdrU detection. In this application, the EdU contains the alkyne and the Alexa Fluor® dye contains the azide. EdU was added to the culture media to a 10 μM final concentration. After a 24 h incubation, spheroids were rinsed in PBS and then fixed. EdU detection, based on a click reaction between EdU and Alexa FLuor® 488 or 594 dye, was performed following manufacturer's instructions.

### Hypoxia detection

Hypoxia detection was performed by using Hypoxyprobe™-1 Plus kit (HPI). Pimonidazole hypochloride (Hypoxyprobe-1)™ was added to the culture media to a 100 μM final concentration for 2 h at 37°C. Pimonidazole forms stable adducts with proteins in hypoxic cells. After fixation, spheroids were processed for frozen sections and pimonidazole adducts were detected by incubating sections with FITC conjugated MAb1 (monoclonal antibody provided, 1/300) for 2 h at 37°C. After PBS washes, DNA was stained using DAPI.

### Pharmacological treatments

Spheroids measuring 350-400 μm in diameter were treated by adding lovastatin (10 μM, Mevinolin, Sigma) or etoposide (1 μM or 5 μM, Sigma) to the culture medium. At the indicated time after treament (24 h or 48 h), spheroids were fixed for 2-3 hrs with formalin (Sigma), then washed with PBS and stored at 4°C.

### Images acquisition and analysis

Fluorescence images from spheroid sections were acquired using a DM5000 (Leica) epifluorescence microscope, fitted with a Roper COOLsnap ES CCD camera coupled using a C-mount 0.63X adapter. Using a 20X objective, the final magnification was 12.5X and allows visualizing entire sections of small and large spheroids in the field of view with image quality high enough for subsequent analyses. Images were processed using Metamorph and ImageJ softwares. To quantify the percentage of Fucci-expressing cells depending on the distance to the spheroid surface, images were analyzed using the Cellomics Technologies software (Compartimental Bioapplication-Thermo Scientific). Briefly, all the nuclei from each section were segmented based on DAPI fluorescence. Then, the x and y coordinates and the fluorescence intensity corresponding to Fucci-Red or Fucci-Green channels were automatically extracted for each individualized object. The same threshold was used to identify Fucci-expressing cells on all the spheroid sections from a single experiment. Numeric values were processed using Microsoft Excel and Prism software packages. For each section, the percentage of positive cells at a given distance was calculated by dividing the number of Fucci-expressing cells by the total number of nuclei included in the indicated range of distance. For each condition, 20 to 30 sections from 8 to 10 spheroids from 2 to 3 independent experiments were analyzed.

## Results

### Growing Capan-2 spheroids display a proliferation gradient

Our first aim was to examine how regionalization of proliferation parameters occurs in Capan-2 multicellular tumor spheroids. To address this issue we selected two representative spheroid growth stages that will subsequently be referred to in this work as small and large spheroids. These stages correspond to spheroids of an average diameter of 300-350 μm and 500-600 μm, respectively. The experiments reported below were performed and strictly restricted to spheroid sections that were identified as closely passing through the spheroid center.

**Figure 1:**
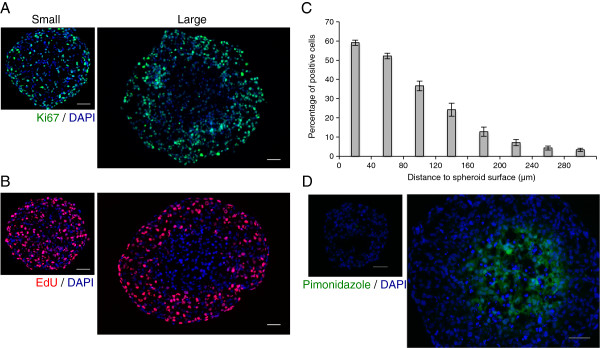
**Proliferation gradient characterization during Capan-2 multicellular tumor spheroids growth.** (**A**) Immunodetection of proliferative cells (Ki-67, green) on a frozen section of a small Capan-2 spheroid (300 μm in diameter, left) or a large Capan-2 spheroid (500 μm in diameter, right). Nuclei are stained with DAPI (blue). Scale bar: 50 μm. (**B**) Visualization of proliferative cells after 24 h of EdU incorporation (red) on a frozen section of a small Capan-2 spheroid or a large spheroid. Nuclei are stained with DAPI (blue). Scale bar: 50 μm. (**C**) Graphic representation of the percentage of proliferative cells after 24 h of EdU incorporation related to the distance to spheroid surface within Capan-2 MCTS measuring 500 μm +/− 20 μm in diameter. Data correspond to the mean +/− SEM of percentage of EdU positive cells from 10 sections similar to the one shown in (**B**) from 4 different spheroids. (**D**) Visualization of the hypoxia by pimonidazole detection (green) on frozen sections from a small spheroid or a large spheroid. Nuclei are stained using DAPI (blue). Scale bar: 50 μm.

We first assessed proliferation using Ki-67 immunofluorescence staining procedure. The Ki-67 antigen is a marker of cell proliferation widely used to evaluate the proliferation index in tissue biopsies [18]. As shown in Figure 1A small spheroids display a homogeneous Ki-67 positive cells distribution pattern, while in contrast on large spheroids Ki-67 positive cells staining is restricted to the outmost cell layers. This first result illustrates the notion of an existing proliferation gradient that is initially absent and develops as multicellular tumor spheroids grow.

To refine this analysis, we sought to visualize the population of cells that are engaged in cell cycle progression and therefore undergoing S-phase replication. To this aim we performed EdU (5-ethynyl-2´-deoxyuridine) incorporation for 24 h. EdU is a nucleoside analog to thymidine that is incorporated in DNA during replication and is therefore a functional marker of actively proliferating cells. As shown in Figure 1B, while in small spheroids staining was observed in the whole volume, a clear regionalization was observed in large spheroids with EdU incorporation restricted to the outmost layers. In such large spheroid quantification of the percentage of EdU positive cells (Figure 1C) as a function of the distance to the spheroid surface displays a progressive decrease of the proportion of proliferative cells from the surface to the center of the spheroid. This experiment shows that approximately 60% of cells have undergone S-phase over the last 24 h and can be considered as actively proliferating in the outer 40 μm of the spheroid. Strikingly, in the same conditions one can also observe a rapid decrease with less than 20% of cells that had undergone DNA replication 150 μm away from the spheroid surface and the absence of any EdU positive cell in the center of the spheroid.

Finally, we also examined whether the observed regionalization of Ki67 and EdU staining matched the hypoxia gradient that progressively takes place in growing spheroids. Indeed, as shown in Figure 1D, while no hypoxia is detected in small spheroids, a strong Pimonidazole staining is observed in large spheroids and its regionalization matches those of the proliferation parameters.

### Cell cycle regionalization within growing Capan-2 spheroids

**Figure 2:**
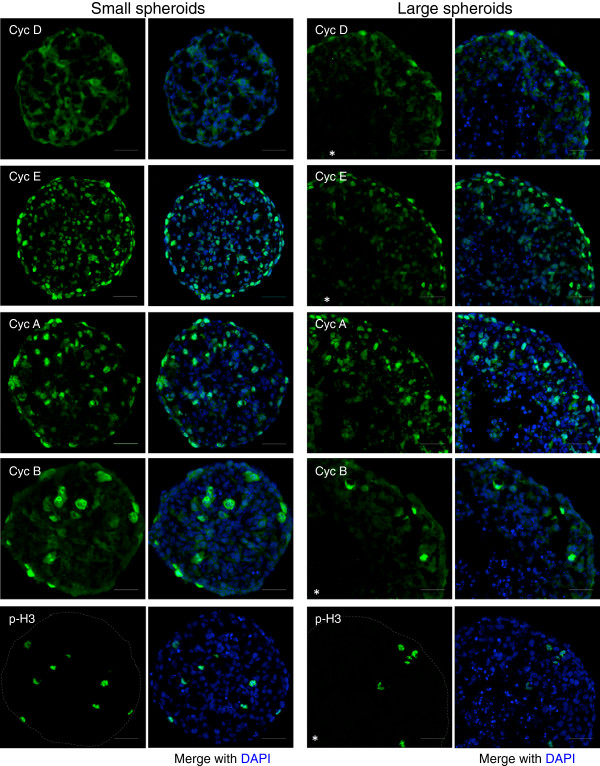
**Expression of cell cycle regulators in small and large Capan-2 spheroids.** Immunofluorescence staining with the indicated antibodies and DAPI performed on small (left column) and large (right column) Capan-2 spheroids Scale bar: 50 μm. The star indicates approximately the center of large spheroids.

The observed regionalization of Ki-67 staining and EdU incorporation in large spheroids indicates that the ability of cells to progress in the cell cycle is dependent on their position to the spheroid surface and suggests that expression of cell cycle regulatory proteins might be dependent on that parameter. We therefore explored systematically cyclins expression, which is representative of the distinct phases of cell cycle, and of phospho-histone H3 to detect mitotic cells on small and large spheroids sections. In Figure 2, from top to bottom are presented staining observed for cyclin D1, cyclin E, cyclin A, cyclin B and phospho-Histone H3.

Cyclin levels are known to mirror the progression of a cell within the cell cycle. As expected, in small spheroids mainly constituted of actively proliferating cells, all four cyclins were highly expressed, with level variations reflecting their position in the cell cycle. In contrast, large and regionalized spheroids displayed an expression pattern of all cyclins that mirrored the proliferation gradient reported above. Finally, detection of phosphorylated histone H3 allowed the detection of mitotic cells that are evenly spread in small spheroids and again restricted to the outer layers in large spheroids.

All together these observations led us next to investigate whether and how cell cycle distribution was a parameter subjected to changes upon spheroid growth and in various layers of a large spheroid. To address that question in a more systematic and quantitative manner than immunostaining detection, we developed spheroid models expressing Fucci (Fluorescence Ubiquitination Cell Cycle Indicators) tools that allow monitoring cell cycle position of a cell [19]. Fucci-red (Cdt1-mKO2) is expressed and stable in G1 cells, while Fucci-green (Geminin-mAG) marks S- and G2-phases. We cloned these constructs and prepared lentiviruses particles that were used to transduce and establish stable Capan-2 cell lines expressing these fluorescent fusion proteins [17]. To validate the cell cycle regulation of these reporters in 3D MCTS, dynamics of Fucci-green and -red expression was examined by co-immunostaining with cyclin antibodies on small and large spheroids. No Fucci-green positive cells (i.e. S-G2-M cells) were found to express the G1-phase cyclins D or E, and no Fucci-red positive cells (i.e. G1 cells) were found to express the G2-phase cyclins A or B (see Additional file 1).

**Figure 3:**
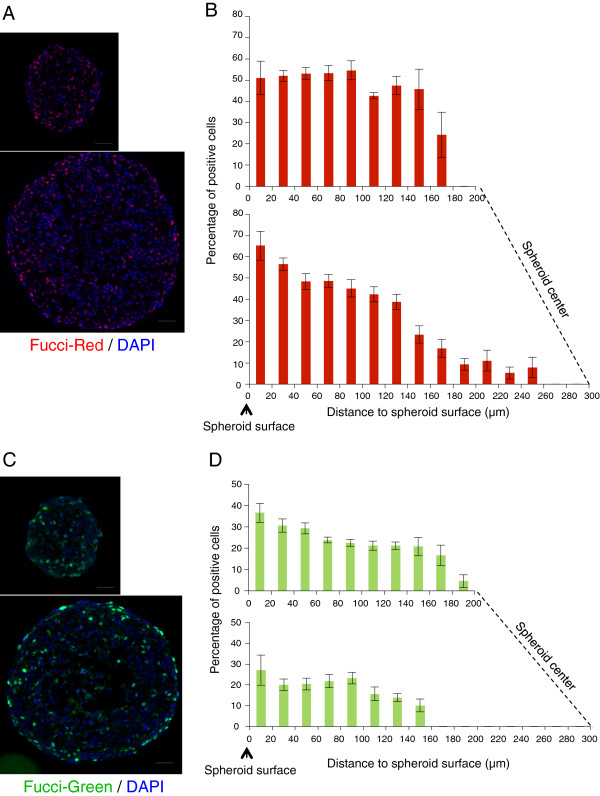
**Cell cycle distribution in Capan-2 spheroids expressing the Fucci-Red or -Green reporters.** (**A, C**): Visualization of the G1 and S-G2 cells on a frozen section of a large Capan-2 spheroid expressing the Fucci-red and Fucci-green reporters respectively. Nuclei are stained with DAPI (blue). Scale bar: 50 μm. (**B, D**): Graphic representations of the percentage of cells expressing the Fucci-red or Fucci-green reporters related to the distance to spheroid surface within Capan-2 MCTS measuring 340 μm +/− 20 μm (top) or 580 μm +/− 20 μm (bottom) in diameter. The relative position of the spheroid center is indicated. Data correspond to the mean+/−SEM of percentage of positive cells on 7-10 sections from 3-4 different spheroids.

Figure 3A and 3C present typical wide field fluorescence microscopy images of spheroids sections illustrating the distribution of Fucci-red and Fucci-green in small and large spheroids. As shown, an even distribution of both markers is observed in small spheroids, while a clear gradient of both Fucci-red and Fucci-green is detected in large spheroids. These results were quantified by determining the percentage of Fucci-red and Fucci-green positive cells as a function of the distance to the spheroid surface. These graphs drawn at the same scale for small and large spheroids clearly illustrate the fact that G1-phase cells are evenly spread in small spheroids (average 50% of positive cells), while in large spheroids a gradient is observed with a noticeable decay approximately 150 μm away from the cell surface (Figure 3B). The percentage of Fucci green cells (i.e. S, G2 and mitosis) is less homogenously distributed in small spheroids with a progressive decrease, while in large spheroids the percentage of positive cells is reduced and no detectable Fucci-green positive cells are observed approximately 120-150 μm away from the surface (Figure 3D).

Altogether, these data indicate that the proliferative zone of a spheroid is restricted to the outer 150 μm where Ki-67 staining is positive and EdU incorporation occurs. Indeed cells are apparently able to efficient cell cycle commitment and to proceed in S-phase in this area only, confirming the above results. These experiments further underline the importance of the growth regionalization aspect occurring upon MCTS growth when investigating cell cycle checkpoint in 3D using a drug that impairs cell cycle progression.

### Lovastatin-induced cell cycle arrest in G1

Lovastatin inhibition of mevalonate synthesis results in farnesyl- and geranylgeranyl-pyrophosphate deprivation that ultimately causes an inactivation of Ras and Rho. It has been demonstrated that up-regulation of p27 mediated by the reduction of Rho geranylgeranylation is responsible for the inhibition of CDK2 activity and consequently for the arrest of the cell cycle at the G1-phase [20,21]. Accordingly, we observed that treatment of a 2D monolayer culture of Capan-2 cells expressing Fucci with lovastatin (60 μM) results in an efficient cell cycle arrest in G1 (85%) with about 90% of Fucci-red and 10% of Fucci-green cells (see Additional file 2). In order to validate that Fucci-red or Fucci-green expressing Capan-2 MCTS could allow evaluating an arrest of the cell cycle at the G1-phase, we analyzed the variation of Fucci reporters’ expression induced by a Lovastatin treatment.

**Figure 4:**
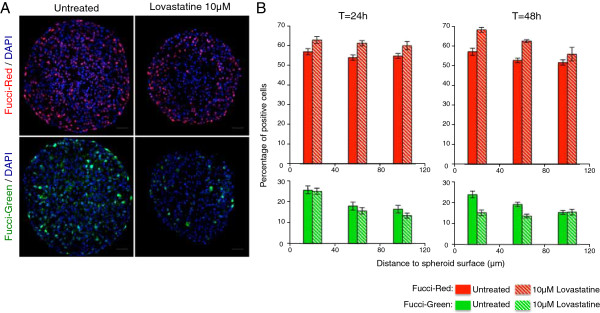
**Lovastatin-induced cell cycle arrest in G1.** (**A**): Capan-2 spheroids treated or not with 10 μM lovastatin for 48 h. Visualization of Fucci-red and Fucci-green expressing cells. Nuclei are stained with DAPI (blue). Scale bar: 50 μm. (**B**) Capan-2 spheroids treated or not with 10 μM lovastatin for 24 or 48 h. Quantification of the percentage of Fucci-red and Fucci-green expressing cells as a function of the distance to the spheroid surface.

When applied on spheroids, we find that lovastatin treatment impairs growth in a dose-dependent manner with an IC50 of 5 μM and induces cell death with spheroid collapse at high concentration. We therefore examined the effect of 5 μM (data not shown) and 10 μM lovastatin after 24 h and 48 h treatment of Fucci-red or -green spheroids. As illustrated with the micrographs shown in Figure 4A and the quantification shown in Figure 4B, the percentage of Fucci-green cells decreases over time while an increase in Fucci-red positive cells is detected. At 48 h, we observe a nearly 40% reduction of Fucci-green positive in the outmost region of the spheroid and in parallel in the same region the percentage of Fucci-red positive cells increases from 57% to 68%. Thus, albeit about 15% of Fucci-green cells are still detected after 48 h, a high level of Fucci-red positive cells indicative of an arrest in G1 is observed in the outmost proliferative layers.

### Monitoring G2/M checkpoint activation in MCTS treated with etoposide

Etoposide (VP-16) is a topoisomerase inhibitor that can be used to induce DNA damage and activate cell cycle checkpoint. As this is the case with many other cell lines, a one hour treatment with 40 μM etoposide results after a 24 h release in the massive accumulation of Capan-2 cells at the G2/M checkpoint that can be detected either by flow cytometry or by the observation of the accumulation of 90% of Fucci-green positive cells with a very low residual percentage of Fucci-red (see Additional file 2).

**Figure 5:**
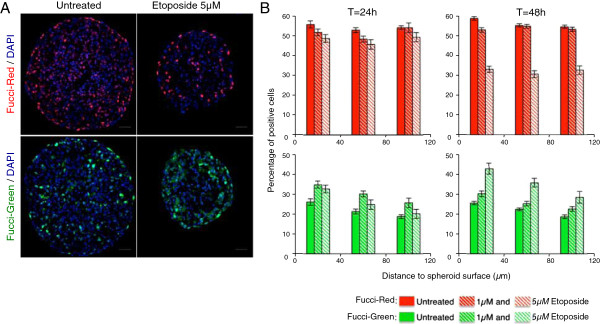
**G2/M checkpoint activation in MCTS treated with etoposide.** (**A**): Capan-2 spheroids treated or not with 5 μM etoposide for 48 h. Visualization of Fucci-red and Fucci-green expressing cells. Nuclei are stained with DAPI (blue). Scale bar: 50 μm. (**B**) Capan-2 spheroids treated or not with 1 μM or 5 μM etoposide for 24 or 48 h. Quantification of the percentage of Fucci-red and Fucci-green expressing cells as function of the distance to the spheroid surface. For each condition, data correspond to the mean+/−SEM of percentage of positive cells on 10-30 sections from 2 or 3 independent experiments with 5 to 10 different spheroids each.

Since it is not technically possible to wash away etoposide from a treated spheroid, we used continuous treatment with a 1 μM and 5 μM concentration for 24 h and 48 h. The micrographs of spheroids treated for 48 h (Figure 5A) illustrate the decrease in Fucci-red cells with a concomitant increase in Fucci-green cells percentage. As presented in the quantitation of these experiments shown in Figure 5B, while modestly affected at 24 h, after a 48 h treatment with 5 μM the percentage of Fucci-green positive cells increases by approximately two-fold (25% to 45%) and in parallel the percentage of Fucci-red positive cells drops from 60% to 30%. Strikingly, this effect is observed up to 120 μm deep in the spheroid. Phospho H2AX staining was used to detect DNA damage and confirmed the presence of numerous positive cells in the whole volume of the spheroid, thus indicating that etoposide has penetrated in the spheroid (see Additional file 3). One can therefore conclude that etoposide treatment of 3D MCTS efficiently activates a DNA damage checkpoint resulting in a global change in cell cycle distribution of the outmost layers of the spheroid.

### Cell cycle arrest in G1 and G2 in response to EGF removal

In order to investigate growth factor starvation effect on cell cycle parameters in MCTS we referred to our recent observation of the dependence of Capan-2 spheroid growth on the presence of EGF [17]. In that context, EGF deprivation effect on cell cycle progression can only be explored within 3D MCTS as Capan-2 cells proliferate in the absence of EGF when grown as 2D monolayer. Capan-2 Fucci-red or -green expressing spheroids grown in DMEM/F12 media were deprived of EGF in the presence of 10% serum and monitored over 6 days. EdU incorporation was performed every day for a 24 h duration to assess the ability of the cells to enter S-phase during that time.

**Figure 6:**
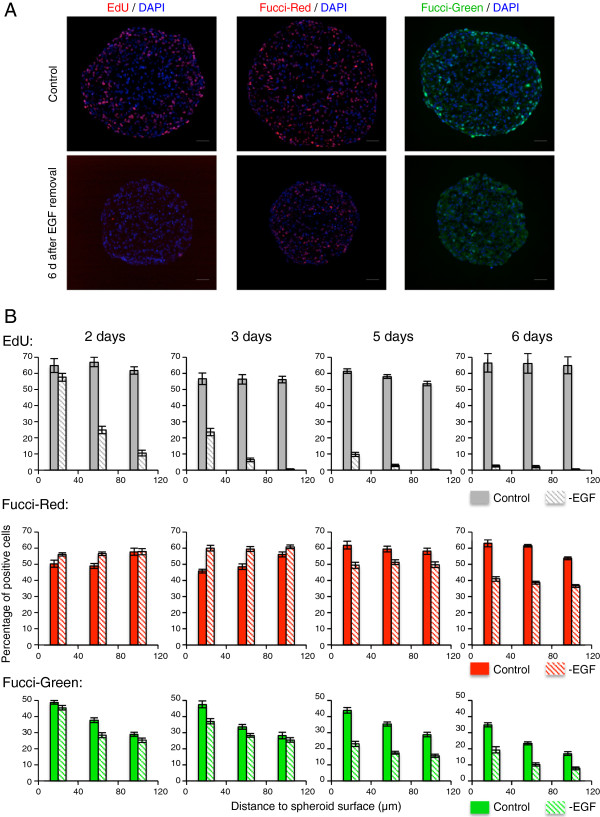
**Cell cycle arrest in G1 and G2 in response to EGF removal.** (**A**): Capan-2 spheroids control or 6 days after EGF removal. Visualization of EdU incorporation (left), Fucci-red (middle) and Fucci-green (right) expressing cells. Nuclei are stained with DAPI (blue). EdU has been incorporated for 24 h. Scale bar: 50 μm. (**B**) Quantification of the percentage of EdU (top), Fucci-red (middle) and Fucci-green (bottom) expressing cells as a function of the distance to the spheroid surface. 0-40 μm, 40-80 μm and 80-120 μm intervals are considered. For each condition, data correspond to the mean+/−SEM of percentage of positive cells on 10-30 sections from 2 or 3 independent experiments with 5 to 10 different spheroids each.

As shown in Figure 6A (left panels) EdU incorporation is totally abolished after 6 days indicating that most cells are likely in a quiescent stage *i.e.* in a prolonged cell cycle arrest. Quantification of the evolution of EdU incorporation overtime as a function of the distance to the cell surface is presented in Figure 6B (top). As shown, there is virtually no EdU incorporation after 5 days and a major decrease is already detected in EGF-deprived spheroids at day 2 in the deepest layers and at day 3 on the outer layers.

According to the classical view of cell cycle regulation such result suggests that upon growth factor removal cycling cells are unable to progress in G1, do not pass the restriction point and are arrested in G1 prior to enter a quiescent state. We examined this hypothesis by looking at the distribution of the percentage of Fucci-red and Fucci-green cells in the proliferative zone of the spheroid during this 6 days experiment. As presented in the micrographs shown in Figure 6A (middle and right panels) both the percentage of Fucci-red and Fucci-green positive cells are diminished after 6 days. The quantification presented in Figure 6B shows that percentage of Fucci-green (i.e. S and G2 cells) progressively dropped and is decreased by a two-fold factor after 6 days. However, unexpectedly about 15% of positive Fucci-green cells are still detected at day 6, while as indicated above EdU incorporation is totally abolished. The evolution of the percentage of Fucci-red positive cells is different. During the first three days, this percentage increases as compared to control spheroid, indicating that cells are accumulating in G1-phase. However, at days 5 and 6 we observe a major decrease in the percentage of Fucci-red positive cells, illustrating the entry of the cells in a quiescent stage accompanied by the loss of Fucci-red expression, that reproduces the decrease of the level of the endogenous Cdt1 protein in the same conditions (data not shown).

Our results strongly suggest that epidermal growth factor removal slows down cell cycle progression not only in G1 but also in G2. The progressive changes in Fucci-green and -red cells percentage thus would likely reflect a combined effect and its progressive consequences on the cell cycle distribution.

## Discussion

This work presents, to our knowledge, the first description of cell cycle parameters in 3D MCTS from the Capan-2 pancreatic adenocarcinoma cell line. We demonstrate that classical immunological reagents, as well as engineered cell lines with Fucci reporters can be used to precisely describe in "cell cycle words" the complex regionalization that is observed upon spheroid growth *in situ*. We showed that in this Capan-2 spheroid model, the actively proliferating cells are restricted to the outer 150 μm of the spheroid. The observed gradient is similar to reported proliferation gradient on other cell types spheroid models [2,22]. However the indicated limit from the surface can vary upon the size of the spheroid, the relative cell packing density and the cell type [2].

The decrease of the percentage of EdU positive cells and the absence of proliferating cells at the center of the spheroid are likely to reflect the fact that as function of the distance to the spheroid surface (i.e. away from nutrients, oxygen, …) cells are increasingly prevented to pass through the restriction point and to commit into the cell cycle, resulting in a progressive increase in G1 duration and consequently in the enlargement of cell cycle duration. This could be related to the persistence of G1/Fucci-red expressing cells in inner regions where there is no more S-G2/Fucci-green expressing cell. Longer EdU incorporation (up to 48 h) resulted only in a slight (10%) increase in the percentage of EdU positive cells, thus indicating that only a very minor fraction of cells has an overall cell cycle duration from 24 to 48 h. Additionally, these data also suggest that quiescence associated with loss of Fucci-red positivity, according to Xouri et al. [23], is probably progressively occurring in cells located from 120 μm to the center of the spheroid. This progressive G1 enlargement and entry into quiescence is probably associated with increase Cyclin-dependent kinase inhibitors (CKI) expression as suggested by LaRue et al. on different spheroid models [2,22]. Unfortunately, none of the antibodies directed against CKIs has given a satisfying signal in our experimental conditions.

We reported three examples of treatment that result in quite different behavior of the spheroids and illustrate that major discrepancies could be observed between 2D and 3D evaluation. The limited and regionalized effects of lovastatin at tolerable concentration might reflect a lovastatin penetration issue, as this has been reported with other compounds [24] and is known as a limitation of the model. Nevertheless, one might consider the penetration in 3D MCTS as an essential parameter to predict the ability of a candidate drug to penetrate in the deepest domains of tumor microregions. Another possible explanation is that the concentration of lovastatin needed to observe a massive cell cycle effect upon depletion of mevalonate is higher than the concentration leading to the deleterious effects associated with inhibition of HMG-CoA reductase in 3D culture. Data obtained with the activation of the DNA damage checkpoint by etoposide provide an example of detectable modification of cell cycle suggesting that Capan-2 Fucci expressing spheroid models could be used to detect or to investigate the effect of a novel DNA damaging compound. EGF deprivation sensitivity of a Capan-2 spheroid and its effect on cell cycle distribution could not have been predicted on a 2D culture as cells are no dependent on this growth factor when grown as a monolayer. Our results showed that growth arrest of Capan-2 spheroids upon EGF removal relies on a cell cycle progression slowdown in G1 but also G2. It has been proposed that EGFR signaling might play a role in the G2/M phase of the cell cycle in human tumor cell overexpressing EGFR [25]. Our observations suggest that, when grown in 3D, Capan-2 cells are prone to arrest or slow down their cell cycle in G2 in response to EGF deprivation.

## Conclusion

One of the main lessons from this study is that one cannot simplistically extrapolate cell cycle effects observed in 2D culture to 3D models such as spheroids. Making for instance the comparison of the etoposide effect in both models would suggest that the observed result in 3D is quite moderate. However, our study warns us on the fact that we should completely rethink the way we interpreted and analyze the effect of a given drug on the cell cycle. A tumor, or a tumor model such as a MCTS is not a population of exponentially growing cells that cycle from G1 to S, G2 and mitosis without any constraint. In spheroid, the major parameters that have to be taken into consideration are regionalization and above all heterogeneity of the cell cycle parameters and distribution within the outermost layers constituting the proliferating zone.

## Abbreviations

MCTS: MultiCellular tumor spheroid;Fucci: Fluorescence Ubiquitination cell cycle indicators;EGF: Epidermal growth factor.

## Competing interest

The authors declare that they have no competing interests.

## Authors’ contributions

JL, CF, OM and MC performed the experiments. VL and BD initiated the project, contributed with data analysis and wrote the manuscript. All authors read and approved the final manuscript.

## Pre-publication history

The pre-publication history for this paper can be accessed here:

http://www.biomedcentral.com/1471-2407/13/73/prepub

## Supplementary Material

Additional file 1 **Characterization of Fucci-red and Fucci-green expressing spheroids.** Immunostaining of Fucci-red and Fucci-green expressing cells with antibodies directed against cyclin D, cyclin E, cyclin A and cyclin B on small (A) or large (B) spheroids. Click here for file

Additional file 2 **Characterization of the effects of lovastatin and etoposide on Capan-2 cells lines expressing Fucci reporters.** Capan-2 cell lines cultured as monolayers and treated or not with lovastatin (60 μM, 48 h) or etoposide (40 μM, 1 h). (A) Visualization of Fucci-red and Fucci-green expressing cells. (B) Flow cytometry analysis of DNA content after DRAQ5 staining. (C) Quantification of the percentage of Fucci-red and Fucci-green expressing cells. Click here for file

Additional file 3 **Etoposide treatment-induced DNA damages in MCTS.** Capan-2 MCTS were treated or not with 5 μM etoposide for 48 h. The genotoxic effect of etoposide was analyzed by immunodetection of the phosphorylation of the H2AX histone variant (red) on frozen sections. Nuclei are stained with DAPI (blue). Scale bar: 50 μm. Click here for file

## References

[B1] Dubessy C Merlin JM Marchal C Guillemin F Spheroids in radiobiology and photodynamic therapy Crit Rev Oncol Hematol 2000 36 2-3 179 192 10.1016/S1040-8428(00)00085-8 11033305

[B2] Sutherland RM Cell and environment interactions in tumor microregions: the multicell spheroid model Science 1988 240 4849 177 184 10.1126/science.2451290 2451290

[B3] Friedrich J Ebner R Kunz-Schughart LA Experimental anti-tumor therapy in 3-D: spheroids-old hat or new challenge? Int J Radiat Biol 2007 83 11-12 849 871 10.1080/09553000701727531 18058370

[B4] Hirschhaeuser F Menne H Dittfeld C West J Mueller-Klieser W Kunz-Schughart LA Multicellular tumor spheroids: an underestimated tool is catching up again J Biotechnol 2010 148 1 3 15 10.1016/j.jbiotec.2010.01.012 20097238

[B5] Desoize B Jardillier J Multicellular resistance: a paradigm for clinical resistance? Crit Rev Oncol Hematol 2000 36 2-3 193 207 10.1016/S1040-8428(00)00086-X 11033306

[B6] Kunz-Schughart LA Freyer JP Hofstaedter F Ebner R The use of 3-D cultures for high-throughput screening: the multicellular spheroid model J Biomol Screen 2004 9 4 273 285 10.1177/1087057104265040 15191644

[B7] Dai Y Grant S New insights into checkpoint kinase 1 in the DNA damage response signaling network Clin Cancer Res 2010 16 2 376 383 10.1158/1078-0432.CCR-09-1029 20068082 PMC2939735

[B8] Malumbres M Barbacid M Cell cycle, CDKs and cancer: a changing paradigm Nat Rev Cancer 2009 9 3 153 166 19238148 10.1038/nrc2602

[B9] Degenhardt Y Lampkin T Targeting Polo-like kinase in cancer therapy Clin Cancer Res 2010 16 2 384 389 10.1158/1078-0432.CCR-09-1380 20068088

[B10] Hanahan D Weinberg RA Hallmarks of cancer: the next generation Cell 2011 144 5 646 674 10.1016/j.cell.2011.02.013 21376230

[B11] Carpinelli P Moll J Aurora kinases and their inhibitors: more than one target and one drug Adv Exp Med Biol 2008 610 54 73 10.1007/978-0-387-73898-7_5 18593015

[B12] Cicenas J Valius M The CDK inhibitors in cancer research and therapy J Cancer Res Clin Oncol 2011 137 10 1409 1418 10.1007/s00432-011-1039-4 21877198 PMC11827940

[B13] Lazo JS Wipf P Is Cdc25 a druggable target? Anticancer Agents Med Chem 2008 8 8 837 842 10.2174/187152008786847738 19075566 PMC2752834

[B14] Vassilev LT Cell cycle synchronization at the G2/M phase border by reversible inhibition of CDK1 Cell Cycle 2006 5 22 2555 2556 10.4161/cc.5.22.3463 17172841

[B15] Carrassa L Damia G Unleashing Chk1 in cancer therapy Cell Cycle 2011 10 13 2121 2128 10.4161/cc.10.13.16398 21610326

[B16] Ma CX Janetka JW Piwnica-Worms H Death by releasing the breaks: CHK1 inhibitors as cancer therapeutics Trends Mol Med 2011 17 2 88 96 10.1016/j.molmed.2010.10.009 21087899 PMC6905465

[B17] Dufau I Frongia C Sicard F Dedieu L Cordelier P Ausseil F Ducommun B Valette A Multicellular Tumor Spheroid model to evaluate spatio-temporal dynamics effect of chemotherapeutics. Application to the gemcitabine / CHK1 inhibitor combination in pancreatic cancer BMC Cancer 2012 12 1 15 10.1186/1471-2407-12-15 22244109 PMC3280152

[B18] Yerushalmi R Woods R Ravdin PM Hayes MM Gelmon KA Ki67 in breast cancer: prognostic and predictive potential Lancet Oncol 2010 11 2 174 183 10.1016/S1470-2045(09)70262-1 20152769

[B19] Sakaue-Sawano A Kurokawa H Morimura T Hanyu A Hama H Osawa H Kashiwagi S Fukami K Miyata T Miyoshi H Visualizing spatiotemporal dynamics of multicellular cell-cycle progression Cell 2008 132 3 487 498 10.1016/j.cell.2007.12.033 18267078

[B20] Javanmoghadam-Kamrani S Keyomarsi K Synchronization of the cell cycle using lovastatin Cell Cycle 2008 7 15 2434 2440 18677105 10.4161/cc.6364

[B21] Zhong WB Hsu SP Ho PY Liang YC Chang TC Lee WS Lovastatin inhibits proliferation of anaplastic thyroid cancer cells through up-regulation of p27 by interfering with the Rho/ROCK-mediated pathway Biochem Pharmacol 2011 82 11 1663 1672 10.1016/j.bcp.2011.08.021 21907187

[B22] LaRue KE Khalil M Freyer JP Microenvironmental regulation of proliferation in multicellular spheroids is mediated through differential expression of cyclin-dependent kinase inhibitors Cancer Res 2004 64 5 1621 1631 10.1158/0008-5472.CAN-2902-2 14996720

[B23] Xouri G Lygerou Z Nishitani H Pachnis V Nurse P Taraviras S Cdt1 and geminin are down-regulated upon cell cycle exit and are over-expressed in cancer-derived cell lines Eur J Biochem 2004 271 16 3368 3378 10.1111/j.1432-1033.2004.04271.x 15291814

[B24] Frongia C Lorenzo C Gianni F Prevost GP Ducommun B Lobjois V 3D imaging of the response to CDC25 inhibition in multicellular spheroids Cancer Biol Ther 2009 8 23 2230 2236 19823027 10.4161/cbt.8.23.9984

[B25] Walker F Zhang HH Burgess AW Identification of a novel EGF-sensitive cell cycle checkpoint Exp Cell Res 2007 313 3 511 526 10.1016/j.yexcr.2006.10.026 17157295

