# Supplementary material for: Multicellular tumor spheroid models to explore cell cycle checkpoints in 3D
Source: BMC Cancer. 2013 Feb 8;13:73. doi: 10.1186/1471-2407-13-73 (PMC3598667; doi:10.1186/1471-2407-13-73)
Supplement: Additional file 1 — Characterization of Fucci-red and Fucci-green expressing spheroids. Immunostaining of Fucci-red and Fucci-green expressing cells with antibodies directed against cyclin D, cyclin E, cyclin A and cyclin B on small (A) or large (B) spheroids. [file 1471-2407-13-73-S1.pdf]

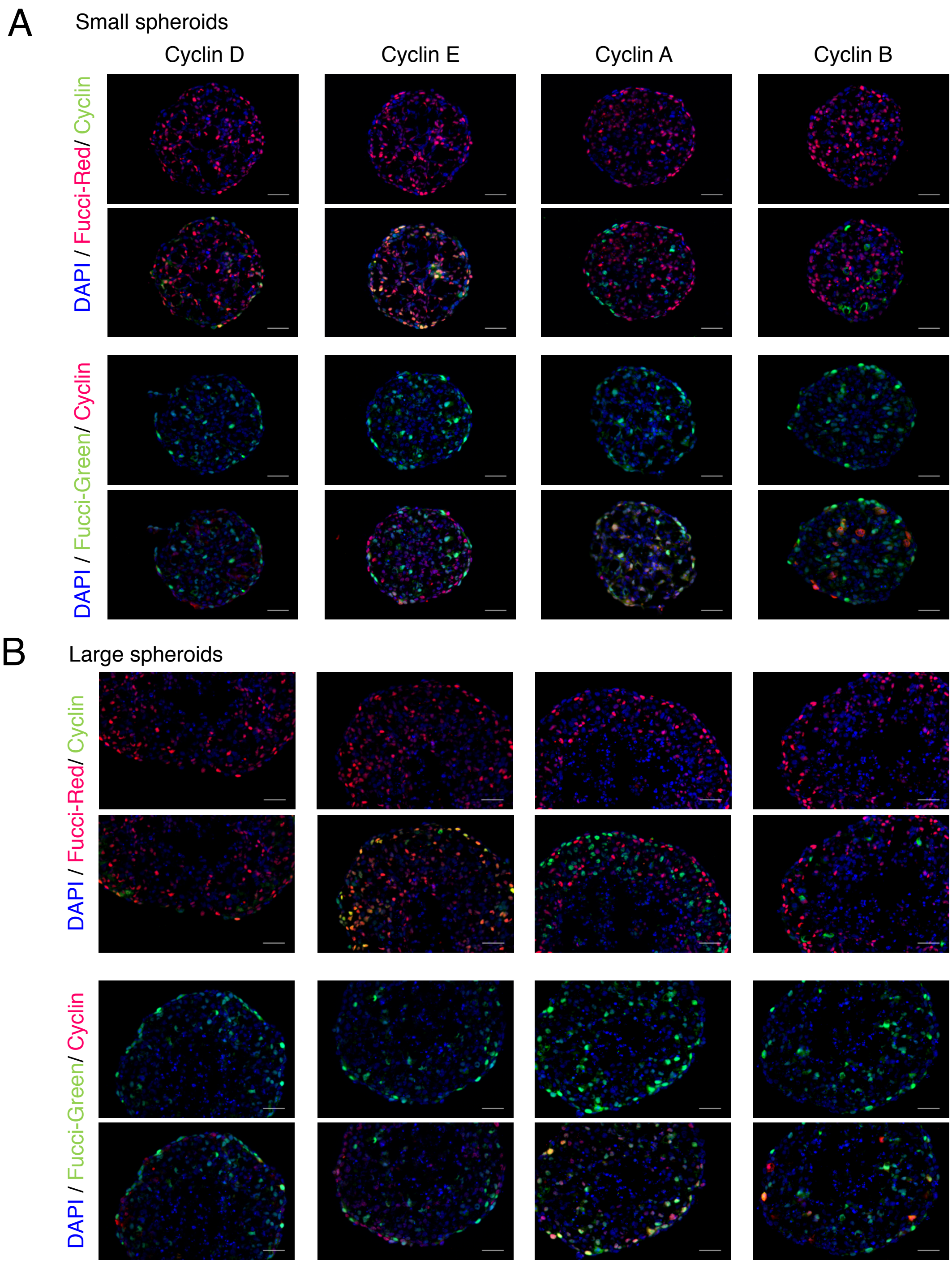

Additional file 1.

Characterization of Fucci-red and Fucci-green expressing spheroids. Immunostaining of Fucci-red and Fucci-green expressing cells with antibodies directed against cyclin D, cyclin E, cyclin A and cyclin B on small (A) and large (B) spheroids.
