# Supplementary material for: Multicellular tumor spheroid models to explore cell cycle checkpoints in 3D
Source: BMC Cancer. 2013 Feb 8;13:73. doi: 10.1186/1471-2407-13-73 (PMC3598667; doi:10.1186/1471-2407-13-73)
Supplement: Additional file 2 — Characterization of the effects of lovastatin and etoposide on Capan-2 cells lines expressing Fucci reporters. Capan-2 cell lines cultured as monolayers and treated or not with lovastatin (60 μM, 48 h) or etoposide (40 μM, 1 h). (A) Visualization of Fucci-red and Fucci-green expressing cells. (B) Flow cytometry analysis of DNA content after DRAQ5 staining. (C) Quantification of the percentage of Fucci-red and Fucci-green expressing cells. [file 1471-2407-13-73-S2.pdf]

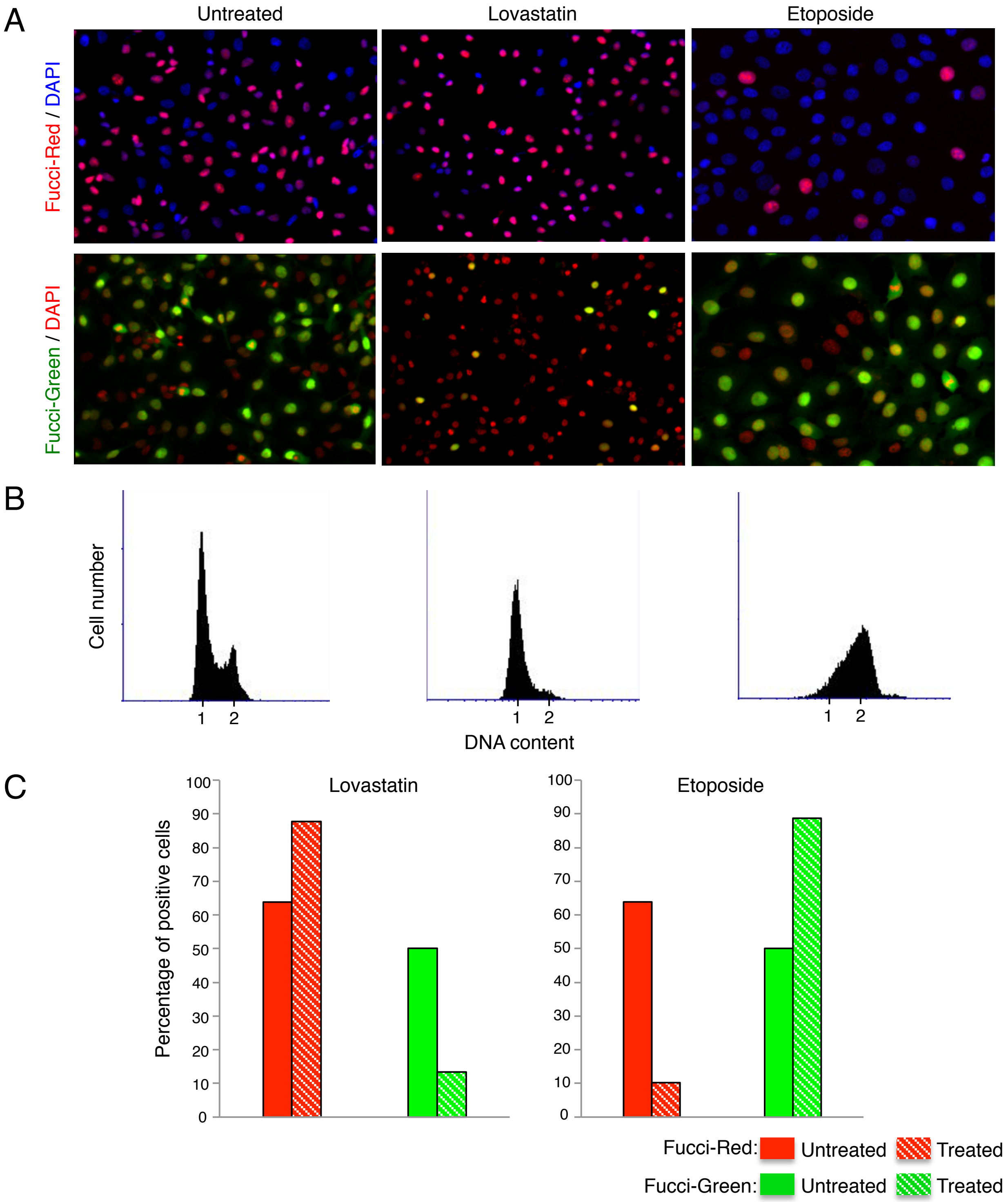

Additional file 2.

Characterization of the effects of lovastatin and etoposide on Capan-2 cells lines expressing Fucci reporters. Capan-2 cell lines cultured as monolayers and treated or not with lovastatin (60 μM, 48 hours) or etoposide (40μM, 1 hour). (A) Visualization of Fucci-red and Fucci-green expressing cells. (B) Flow cytometry analysis of DNA content after DRAQ5 staining.
(C) Quantification of the percentage of Fucci-red and Fucci-green expressing cells.
