# Supplementary material for: Multicellular tumor spheroid models to explore cell cycle checkpoints in 3D
Source: BMC Cancer. 2013 Feb 8;13:73. doi: 10.1186/1471-2407-13-73 (PMC3598667; doi:10.1186/1471-2407-13-73)
Supplement: Additional file 3 — Etoposide treatment-induced DNA damages in MCTS. Capan-2 MCTS were treated or not with 5 μM etoposide for 48 h. The genotoxic effect of etoposide was analyzed by immunodetection of the phosphorylation of the H2AX histone variant (red) on frozen sections. Nuclei are stained with DAPI (blue). Scale bar: 50 μm. [file 1471-2407-13-73-S3.pdf]

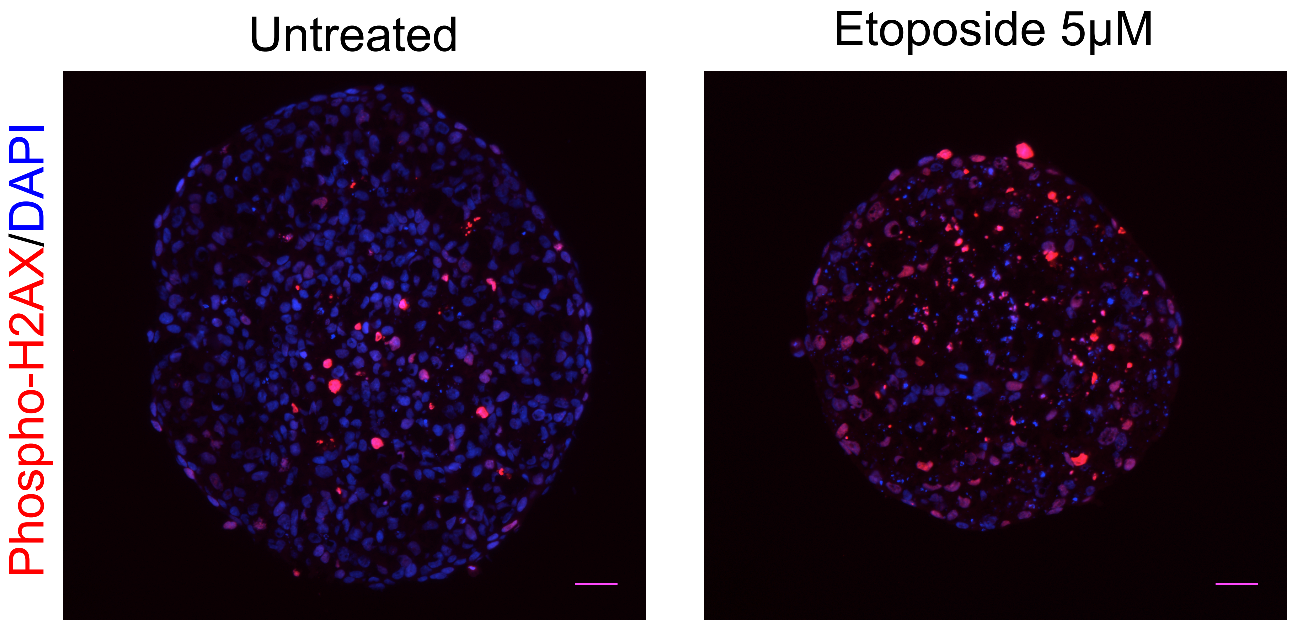

Additional file 3.

Etoposide treatment-induced DNA damage in MCTS.

Capan-2 MCTS were treated or not with 5µM etoposide for 48 hours. The genotoxic effect of etoposide was analyzed by immunodetection of the phosphorylation of the H2AX histone variant (red) on frozen sections. Nuclei are stained with DAPI (blue). Scale bar: 50µm
